# “We bleed for our community:” A qualitative exploration of the implementation of a pragmatic weight gain prevention trial from the perspectives of community health center professionals

**DOI:** 10.1186/s12889-023-15574-2

**Published:** 2023-04-14

**Authors:** Miriam B. Berger, Miriam Chisholm, Hailey N. Miller, Sandy Askew, Melissa C. Kay, Gary G. Bennett

**Affiliations:** 1 Duke Digital Health Science Center, Duke University, 417 Chapel Drive Room 048, Campus Box 90086, Durham, NC 27708-0086 USA; 2 School of Nursing, Duke University, 307 Trent Drive, Durham, NC 27710 USA; 3 Department of Pediatrics, Duke University, 3116 N. Duke Street, Room 1029, 27704 Durham, NC USA; 4 Department of Psychology and Neuroscience, Duke University, 222 Reuben-Cooke, Durham, NC 27708 USA

**Keywords:** Qualitative, Pragmatic trial, Obesity, Community health center, Stakeholder

## Abstract

**Background:**

Clinical trial implementation continues to shift toward pragmatic design, with the goal of increasing future adoption in clinical practice. Yet, few pragmatic trials within clinical settings have qualitatively assessed stakeholder input, especially from those most impacted by research implementation and outcomes, i.e., providers and staff. Within this context, we conducted a qualitative study of the implementation of a pragmatic digital health obesity trial with employees at a Federally qualified health center (FQHC) network in central North Carolina.

**Methods:**

Participant recruitment was conducted through purposive sampling of FQHC employees from a variety of backgrounds. Two researchers conducted semi-structured qualitative interviews and collected demographic data. Interviews were digitally recorded, professionally transcribed and double-coded by two independent researchers using NVivo 12. Coding discrepancies were reviewed by a third researcher until intercoder consensus was reached. Responses were compared within and across participants to elucidate emergent themes.

**Results:**

Eighteen qualitative interviews were conducted, of whom 39% provided direct medical care to patients and 44% worked at the FQHC for at least seven years. Results illuminated the challenges and successes of a pragmatically designed obesity treatment intervention within the community that serves medically vulnerable patients. Although limited time and staffing shortages may have challenged recruitment processes, respondents described early buy-in from leadership; an alignment of organizational and research goals; and consideration of patient needs as facilitators to implementation. Respondents also described the need for personnel power to sustain novel research interventions and considerations of health center resource constraints.

**Conclusions:**

Results from this study contribute to the limited literature on pragmatic trials utilizing qualitative methods, particularly in community-based obesity treatment. To continue to merge the gaps between research implementation and clinical care, qualitative assessments that solicit stakeholder input are needed within pragmatic trial design. For maximum impact, researchers may wish to solicit input from a variety of professionals at trial onset and ensure that shared common goals and open collaboration between all partners is maintained throughout the trial.

**Trial Registration:**

This trial was registered with ClinicalTrials.gov (NCT03003403) on December 28, 2016.

## Background

Clinical research design has recently moved from controlled research environments to more real-world settings [1]. This increase is in part due to the potential for pragmatic clinical trials to provide an evidentiary basis for interventions in real-world settings [2, 3], thus enhancing their dissemination potential [4]. Despite a great deal of evidence in the obesity treatment space, translation of these approaches into practice, especially within primary care continues to be a challenge [5–8]. Recent pragmatic trials that have focused on weight management within the primary care setting include trials that have tested the effects of an online weight loss program to support provider counseling [9]; electronic health record workflow utilization within large healthcare systems for weight management [10]; and a self-directed lifestyle program for U.S. veterans [11], among others. However, many of the pragmatic trials for weight management within primary care described in the literature lack qualitative research components. This is a critical gap, as qualitative research is integral to optimal pragmatic trial design [12], particularly as a method to solicit input from key stakeholders who work within the clinical setting and with the populations being recruited for - and ultimately impacted by - the research [13].

Of the few weight management pragmatic clinical trials that include qualitative components, they are often conducted with trial patient participants, and do not solicit formal qualitative feedback from providers, staff members, or interventionists involved with trial implementation [14, 15]. Involving key collaborators within the participating healthcare system prior to and throughout implementation may elucidate factors that present barriers of and facilitators to trial implementation and outcomes.

Thus, the purpose of this qualitative study is to explore the barriers of and facilitators to the implementation of a pragmatic clinical weight gain prevention trial called Balance, from the perspectives of healthcare professionals with varying roles in the participating clinical sites. Interview questions solicited input on the challenges and successes of treating obesity within a complex community healthcare network; the weaknesses and strengths of the implementation of a pragmatic weight management trial within a network of Federally qualified health centers (FQHCs); and potential implications for disseminating the intervention to similar settings.

## Methods

### Balance design, setting, and population

Balance was conducted within Piedmont Health Services, Inc. (PHS), a network of 10 Federally qualified health centers (FQHCs) within central North Carolina, many of which are located in rural areas. Each site offers a core set of primary care services that are available in English or Spanish and on a sliding-fee scale basis, with additional site-specific services to address local community needs.

Balance study protocols and intervention components have previously been described in-depth elsewhere. [16, 17] Briefly, Balance is a 12-month pragmatic randomized controlled trial of a weight gain prevention intervention; weight gain prevention is defined as ≤ 3% weight gain over baseline weight at 24-months post-randomization. A total of 443 adult PHS patients with overweight or obesity (BMI ≥ 25) were randomized to: (1) a digital health intervention with personalized behavior change goals; digital self-monitoring with tailored feedback; daily weighing on cellular-connected scales; and responsive remote coaching from registered dietitians; or (2) routine primary care at their health center and health education materials. Aligning with its pragmatic design, all clinical data for trial outcomes were extracted from the PHS electronic health record. All study procedures were approved by the Duke University Campus Institutional Review Board and the Quality Assurance Committee of the PHS Board of Directors.

### Qualitative study recruitment

Recruitment for this qualitative study was conducted in 2019 through purposive sampling of PHS employees from varying roles including: individual site and organizational leaders; medical providers (i.e., physicians, nurse practitioners); care navigators; registered dietitians; and behavioral health providers. Staff members and providers at all five participating community health centers, as well as overall PHS leaders, were invited to participate. PHS leadership sent introductory emails on behalf of the Balance research team to 22 PHS employees from across the organization; an additional nine potential participants were identified by staff members for follow-up. Balance research staff then emailed the employee directly to assess interest and to schedule the verbal informed consent process. To recruit a representative sample from across the organization, Balance staff sent a follow-up email invitation if no reply was received. Participants who provided their consent to participate were asked to complete a 30-45-minute semi-structured in-person or phone interview, based on availability and preference.

### Study instruments

A detailed interview guide was developed and refined with input from research team members and co-investigators. The interviewers were trained by a researcher with qualitative interviewing experience on standardizing the interview process; the purpose of each interview question; and appropriate follow-up with additional probing questions. Key themes assessed included: respondent perceptions of the landscape of obesity treatment within PHS; strengths and weaknesses within the community health system for obesity treatment; and awareness and perceptions of the implementation of the larger pragmatic trial. Because the interview guide was semi-structured in nature, interviewers asked additional probing questions to solicit further clarifying information, as necessary. In addition to the qualitative interview, participants were asked to fill out brief demographic questionnaires regarding their self-identified age, race, ethnicity, job role, educational background, and length of time employed at PHS. Each participant was offered a gift card in appreciation for their time.

### Data analysis

Interviews were digitally recorded and professionally transcribed verbatim. All transcripts were reviewed for accuracy and identifying information was removed prior to analyses. Each interview was double coded for emergent themes by two independent researchers using NVivo 12 (QSR International). Coding was merged and reviewed by a third researcher; discrepancies in coding were discussed until consensus was reached. Responses were compared within and across participants to determine emergent themes. Participant demographics were summarized using means and standard deviations for continuous variables and proportions for categorical variables.

## Results

### Sample

Between April and June 2019, two researchers conducted a total of 18 in-person or phone semi-structured qualitative interviews, representing 58% of those invited to participate (n = 31). At the time of interview, the mean participant age was 36.9 (± 9.7) years, and most respondents identified as female (89%) and non-Latino/a white (67%). Approximately 39% of participants provided direct medical care to patients as physicians or nurse practitioners, and 44% had been employed by PHS for at least seven years. (See Table 1 for participant demographics).

#### Emergent themes

Results from the interviews elucidated the barriers of and facilitators to the implementation of a pragmatic behavior change intervention for obesity treatment within a community healthcare setting that provides patient-centered comprehensive care to primarily medically vulnerable patients (See Table 2 for a summary of emergent themes).

#### Barriers to Balance implementation

Respondents described several barriers to the implementation of Balance within their health system. These included initial concerns about trial referral processes and the potential time burden on providers and other staff; limited health center space for trial activities; and staff shortages and turnover.

Several respondents discussed the challenges of referring interested patients to the trial. An administrative leader commented on provider concerns regarding the potential impact on patient flow and wait times, during already short visits:

“*Buy-in also with medical staff, that was one of the things that was initially a potential problem. [Providers] telling them about the Duke Balance program takes a good five minutes of the visit time. And so, that affects the numbers behind and that affects the way that patients will be waiting longer in the waiting room…It was just the buy-in that was difficult here. And so, providers were… not complaining, but just saying ‘Hey, you know, I’ve got so many things on my plate… I’ve got other patients waiting for me right now.’ And that was a little bit of an issue at first.*”

Another administrative leader also recalled the importance of the initial meetings between the research team and providers to explain the study to providers and how it could potentially help their patients:

“*When we had the first huddles for them [providers] to understand why you guys were there and you were to help rather than being some kind of entity here, just to take our time, basically. You were here for the patients and that’s what matters to us as well. But it took a little while to understand that.*”

In addition to concerns about provider time, limited physical space for regular health center operations, with the addition of trial recruitment activities, was described as a challenge by several participants. One leader commented that *“not being able to provide you guys with a concrete, designated space, I think sometimes made it hard [to implement Balance].”* Several respondents also discussed the unique challenges posed to trial recruitment during an unexpected period of health center renovations in which two health centers merged their staff and site operations into one location. This barrier was summarized by an administrative leader: *“We had very limited amount of space. And that’s three months that you couldn’t do much at our clinic.”*

Staff turnover during the enrollment period of Balance also posed another barrier to trial implementation - particularly the impact on recruitment flow and awareness of the trial. One provider commented:

“*I think we had some staff turnover in the midst of enrollment. I think after the original MA [medical assistant] who had come and gotten the training left, I feel my – the MAs – after that really didn’t know about Balance. And so then the referral process probably got more on me, than on my MAs. There were probably patients that I didn’t think about, only because it hadn’t been on my radar*…*We just had an enormous amount of turnover during the course of this enrollment- provider and staff... When there’s turnover, it’s hard to remind everybody about what the study is about.*”

Staff hiring and departures and the timing of trial introductory meetings with the research team and PHS may have impacted the awareness of the trial. In fact, one-third of participants stated they had no awareness of Balance and therefore could not comment extensively on the challenges with trial implementation.

#### Facilitators to Balance implementation

Despite Balance implementation barriers that were described by participants, the implementation of the trial was positively described by most respondents. They commented on the strong collaboration with the research team and recognition of the health system’s mission to comprehensively serve their medically vulnerable patients.

An administrative leader highlighted the importance of research programs aligning with the health system’s values to serve the community and focus on patient needs:

“*We bleed for our community, and so the thing about our leaders and our staff, we’re here because we care about our community, we’re not here for…a paycheck. But you know, we’re in community health, so part of being in community health is having that desire to really help people, and so, I think being a part of a project like this fits naturally into our mission…And you know, our shared values, open communication is one, you know, collaboration, is one.*”

Another administrative leader echoed the importance of the research partnership to serve the patient-centered mission of PHS:

“*The fact that we are very inclusive of any opportunity for the patients to get better. We do everything that we can for the community and any kind of partnership of that kind, you know is always something we are looking forward to. I think that [Balance] was the right partnership. Because PHS, we are a network of community health centers, and we really take on the responsibility of caring for the ones who cannot find medical care anywhere else.*”

In addition, a medical provider/administrative leader commented on the adaptive recruitment method employed in the trial that addressed initial provider concerns about time and resource constraints by utilizing other staff members to help with recruitment:

“*Nobody can get any funding without serving the underserved. We’re sometimes an afterthought, I think, in a lot of people’s grants. We get approached for a lot of things. I remember meeting specifically with you about okay, how are we going to do this? [You were] very sensitive to providers’ time… Your approach won us over… That is a factor, because once you’re asking everybody in the clinic to participate and help with your study, it can be a burden.”*

Another medical provider also commented on the benefit of a pragmatic trial design to reach health system patients, without adding many additional burdens to healthcare delivery or site operations:

“*I was just excited that I finally had something to offer people right here in the building. I could access it, right here, right now. That’s what most people wouldn’t expect from us, but they get so much right here, right now, with our wraparound services. To also include something to help with their weight was amazing. That’s why I’m so gung-ho about it. I expected them to learn, to learn a little bit about themselves and habits. And I expected them to hopefully feel a little more supported in achieving their health-related, or weight-related goals.*”

An administrative leader summarized the importance of providing opportunities to manage obesity through healthy lifestyle programs, including weight gain prevention:

“*I think that one of the things that you guys educated our providers about is… it doesn’t have to be weight loss. It can be not gaining. That is much more achievable, I think, and with small changes. That’s why that’s more exciting.*”

#### Research implementation and dissemination implications for other clinical care settings

The qualitative interviews with health center staff also elucidated suggestions for similar pragmatic trials to employ. Many of the respondents described the implementation of Balance within the context of the dissemination potential of similar healthy lifestyle interventions in FQHCs to improve patient health and outcomes. Leadership and provider support was highlighted by several respondents as critical for adoption of the intervention, as this provider/leader commented:

“*You just need somebody in leadership who says, will say yes to a research project. It always ends up, it’s more work on the agency, right? You have to have somebody in leadership who understands the potential benefits of research activity for the patients, because that’s where – a lot of people and I don’t blame people say, ‘I just can’t do this. I can’t do this job here at this FQHC and handle researchers. It’s enough for me just to handle patients, patient problems, all the multiple expectations of the Federal government. We are happy to refer to your research program, but we just cannot handle this here.’*”

Other respondents posited that provider support was critical and that the potential benefits to their patients would have to be explained:

“*I guess just buy-in, that it [the intervention] works, that it is helpful for the patients and they’re [patients] the number one priority.*”

Beyond institutional and leadership buy-in, the consideration of staffing challenges was also mentioned multiple times as important to successfully implement and sustain novel interventions especially within resource-constrained settings, as this care manager commented:

“*Just making sure that… we have the staff to be able to do something like this and do it well. ‘Cause I think a lot of times when we get stuff started, it feels like we can get kind of the wheels turning but in order to make it something really long-term sustainably successful…Sometimes I think we struggle with that because of a lack of manpower or just shifting priorities.*”

An administrative leader echoed this and shared that ideal research programs should complement current FQHC operations, without adding additional personnel effort:

“*I’m more interested in those sorts of research programs, where you’re looking at enhancing our services essentially… We just don’t have the overhead. We have one data analyst. Even just [managing patient lists for] recruitment is enough. If we had more infrastructure for research, we could handle more types of research programs.*”

In addition, the benefit of interventions on the health system’s bottom line was identified as important, as changes in the payor system toward value-based care have made evidence-based programs that support health outcomes easier to utilize, as summarized by this provider/leader:

*“As we move into value-based care, the interventions like this that have been tested are going to be much easier to implement, because we can…just say, ‘Well, this is what you need. So, instead of seeing me next month, you’re going to see the nutritionist three times between now and then.’”*

Moreover, partnership between the FQHCs and the research team was described as critical to future successes of implementation. A medical provider/administrative leader highlighted the importance of a collaborative and thoughtful approach to research design that solicits the perspectives of providers and staff in the planning process and respects the wisdom of the implementation site. This was stated as often lacking in academic-community research partnerships:

“*I think that people are shocked when they get here and we give them [academic researchers] those questions, because I think there’s a mentality in academia that people know what’s best and we just need a community site and we’ll bring them something great that they never had access to and we’ll do good for patients. Then I say, ‘What happens after your study? Is that not going to exist anymore?’ That kind of thing. Yeah. The power dynamics. I guess, I know you’re an academic and you’re talking to this doc here who’s been working here for 20 years. I mean, it’s respect. There is [sic] a lot of issues about inclusion of our thinking, that’s before getting to the patients. Please don’t run through us for your patients, right? Talk to us. We’re really the key to your success. We want you to be successful and we want to be able to say, ‘Here are the three things that concern me about your methodology.’*”

## Discussion

To better understand the barriers to and facilitators of the implementation of an obesity-related pragmatic clinical trial within a community health center setting, we conducted in-depth qualitative interviews with 18 professionals representing a variety of positions from across the health system. Respondents described a variety of challenges and strengths alike that impacted the implementation of the trial within their FQHC setting.

Our findings contribute to the limited qualitative assessments conducted with professional stakeholders included in pragmatic clinical trial design. Results from this study echo those from a recent qualitative study by Brooks et al. with physicians regarding their motivation to participate in a larger pragmatic trial for obesity treatment within rural health centers [18]. Respondents in the Brooks study emphasized the need for more treatment options for rurally located patients and the importance of pragmatic trials to fill the gap between ideal and actual clinical care [18]. The study also highlighted the persistent lack of time to address obesity within primary care, despite providers’ desire to help their patients, a resounding theme in our findings. Results from our study echo these conclusions, highlighting the need to align clinical care and research – including those for weight management programs – within primary care.

This qualitative study also supports previous findings of the importance of involving a diverse group of stakeholders within pragmatic clinical trial design [19, 20]. Based on our findings, researchers looking to implement and evaluate novel interventions for obesity and other comorbidities may wish to consider the importance of including providers, leaders and employees throughout the organization early in the planning phase – and not after the trial’s purpose and outcomes have already been determined. Similarly, findings from a recent two-phase qualitative study by Tambor et al. regarding the appropriate level of involvement for clinicians within pragmatic clinical trials concluded that early engagement of a range of stakeholders during trial planning can help avoid common pitfalls to recruitment and implementation [21]. This also aligns with the National Institutes of Health Collaboratory’s shared best practices for pragmatic clinical trials, that engaging leaders and clinicians in the beginning phases – as well as throughout the trial – is important to its eventual success [3]. Our findings also suggest that the awareness and buy-in of staff, particularly medical assistants – is critical to the success of trial implementation.

Respondents in our study who were involved in Balance – and thus aware of its components – highlighted its implementation as a positive research collaboration. They noted the integration of a pragmatic trial within the healthcare system without overburdening or impeding clinical operations. This has been previously noted as an important consideration for researchers. [22, 23] In fact, in their review of the strengths, weaknesses, and challenges of pragmatic trial implementation based on focus groups with providers, the authors concluded that researchers should lead recruitment and follow-up activities with research participants, as many providers are reluctant to participate due to lack of time for research in addition to their clinical responsibilities. [21] This is a critical factor for research teams to consider before partnering with clinical sites for research, especially when trying to maintain a pragmatic approach [24].

This study has some limitations to note. First, despite our success in enrolling participants from across roles within PHS, our sample may not have been reflective of all perspectives across the organization. Second, although patient perspectives are important and were collected as part of post-trial satisfaction surveys, qualitative patient perspectives on the implementation of Balance were beyond the scope of this project. Third, participation in the interviews may have been influenced by selection bias; there were other individuals in the organization whose perspectives would have been helpful but did not respond to requests, and thus our qualitative data are limited to those who volunteered to take part. Specifically, the lack of participation from registered nurses who provide care at the participating health centers may have influenced our results. Finally, staff turnover, identified as an overall challenge within the health center network, may have impacted the scope of knowledge for some participants. In fact, a few staff members in our sample began working at the health system after Balance meetings and trainings had already been conducted and were thus less familiar with the study than other respondents and unable to provide specific feedback on Balance implementation.

Strengths of this study include its addition to the limited qualitative research as part of pragmatic trials, especially among settings serving medically vulnerable populations. Participants highlighted the importance of implementing novel health improvement interventions, such as Balance, and the successes and challenges of conducting pragmatic research within a complex healthcare system that responds to the variety of patient needs. Respondents shared practical suggestions for implementing similar pragmatic research, especially in the design of interventions for medically vulnerable populations and within resource-constrained community healthcare settings. Ensuring transparency, consideration for time and resources; and aligned values and communication between the research and clinical site were all considered by respondents to be critical for successful implementation. This can ultimately facilitate strong collaboration between the healthcare setting and research partners, as has been previously noted. [25] Not only can this improve the strength of the relationship between the various stakeholders, but it may also improve the ease by which the research is executed, and trial outcomes are derived.

## Conclusion

As obesity rates continue to grow and the healthcare payor system moves into value-based care, the need for the implementation and evaluation of pragmatic trials will remain an important component of comprehensive research and clinical care. Findings from this qualitative assessment of the implementation of a pragmatic weight gain prevention trial contribute to the unique perspectives from professionals working within community-based healthcare settings to treat obesity with medically vulnerable patients and emphasize the importance of involving representation from providers, leaders and staff in study design and implementation. As noted in our findings, community healthcare professionals care deeply for their patients and are in touch with their unique needs. For maximum impact, researchers conducting pragmatic trials may wish to employ qualitative research methods – in addition to quantitative ones – with a variety of collaborators within the healthcare setting as part of their implementation plans from the start.

**Table 1:** Participant demographics

Characteristic	N (%) or Mean ± SD (n = 18)
**Age in years, mean ± SD**	36.9 ± 9.7
**Patient Care Roles, N (%)**	
Medical provider	7 (39%)
Dietitian	4 (22%)
Care manager	3 (17%)
Behavioral health consultant	2 (11%)
Exclusively leadership	2 (11%)
**Leadership Roles, N (%)**	
Exclusively patient care	11 (61%)
Corporate leadership	5 (28%)
Site leadership	2 (11%)
**Credentials of Providers, N (%)**	
MD	4 (22%)
NP	2 (11%)
FNP	1 (6%)
RD	4 (22%)
Not applicable	7 (39%)
**Time Employed at PHS, N (%)**	
10 years+	4 (22%)
Between 7 and 9 years	4 (22%)
Between 5 and 7 years	3 (17%)
Between 3 and 5 years	2 (11%)
Between 1 and 3 years	4 (22%)
Less than 1 year	1 (6%)
**Gender, N (%)**	
Female	16 (89%)
Male	2 (11%)
**Combined Race/Ethnicity, N (%)**	
Non-Hispanic White	12 (67%)
Non-Hispanic Asian	2 (11%)
Hispanic White	1 (6%)
Hispanic Multiracial	1 (6%)
Non-Hispanic Black	1 (6%)
Declined to answer	1 (6%)

**Table 2:** Summary of main interview themes

Theme	Barriers	Facilitators
*Balance trial implementation*	• Recruitment challenges at start • Initial provider buy-in • Health center space • Staff turnover • Awareness of trial	• Focus on patients • Fills treatment gap • Consideration of time • Flexibility of research team
*Implications for dissemination in other clinical care settings*	• Personnel power to sustain intervention • Research-related costs and time • Respect for implementation site	• Supports mission and values of implementation site • Consideration of burden on staff • Strong research collaboration from academic partners • Economic impacts

## Data Availability

The datasets generated and/or analyzed during the current study are not publicly available due to the potential loss of confidentiality and disclosure of participant identities but are available from the corresponding author on reasonable request.

## Abbreviations

PHS: Piedmont Health Services, Inc.
FQHC: Federally qualified health center
MA: Medical assistant
